# Supplementary material for: Transcriptomic Insights into Mechanisms of Early Seed Maturation in the Garden Pea (Pisum sativum L.)
Source: Cells. 2020 Mar 23;9(3):779. doi: 10.3390/cells9030779 (PMC7140803; doi:10.3390/cells9030779)

Zhewan−1,25(2)

Global Summary

%DE = 0.24

genes with fdr < 0.2 = 150 (63 + / 87 −)

genes with fdr < 0.1 = 136 (59 + / 77 −)

genes with fdr < 0.05 = 134 (57 + / 77 −)

genes with fdr < 0.01 = 121 (53 + / 68 −)

genes in genesets = 0

<FC> = 0

<t−score> = 0

<p−value> = 0

<fdr> = 0.77

Global Genelist

Rank	ID	log(FC)	p−value	fdr	Metagene	Description
1	TRINITY_DN2	−0.17	2e−16	2e−15	14 x 3	
2	TRINITY_DN1	−9.53	2e−16	2e−15	20 x 1	
3	TRINITY_DN1	3.01	2e−16	2e−15	20 x 20	
4	TRINITY_DN5	0.58	2e−16	2e−15	2 x 20	
5	TRINITY_DN1	0.18	2e−16	2e−15	7 x 20	
6	TRINITY_DN1	−0.19	2e−16	2e−15	10 x 1	
7	TRINITY_DN1	0.17	2e−16	2e−15	7 x 19	
8	TRINITY_DN3	0.22	2e−16	2e−15	1 x 17	
9	TRINITY_DN2	−3.11	2e−16	2e−15	20 x 1	
10	TRINITY_DN2	−0.91	2e−16	2e−15	20 x 1	
11	TRINITY_DN3	−0.17	2e−16	2e−15	19 x 10	
12	TRINITY_DN5	0.17	2e−16	2e−15	1 x 1	
13	TRINITY_DN4	−0.18	2e−16	2e−15	15 x 3	
14	TRINITY_DN4	−0.74	2e−16	2e−15	1 x 1	
15	TRINITY_DN3	−1.16	2e−16	2e−15	20 x 1	
16	TRINITY_DN2	−0.24	2e−16	2e−15	20 x 20	
17	TRINITY_DN4	−0.18	2e−16	2e−15	17 x 9	
18	TRINITY_DN4	−0.61	2e−16	2e−15	1 x 1	
19	TRINITY_DN1	0.17	2e−16	2e−15	6 x 19	
20	TRINITY_DN1	−0.2	2e−16	2e−15	16 x 5	

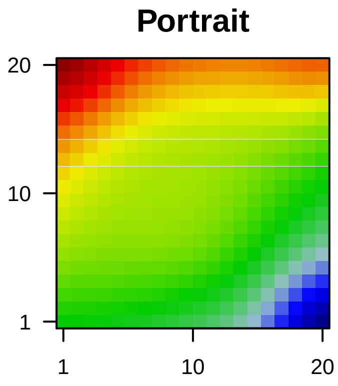

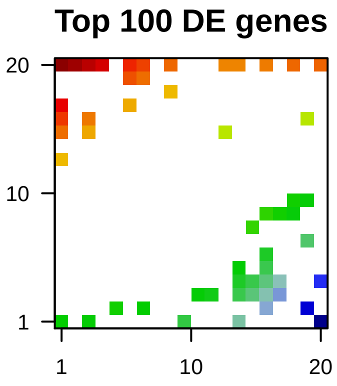

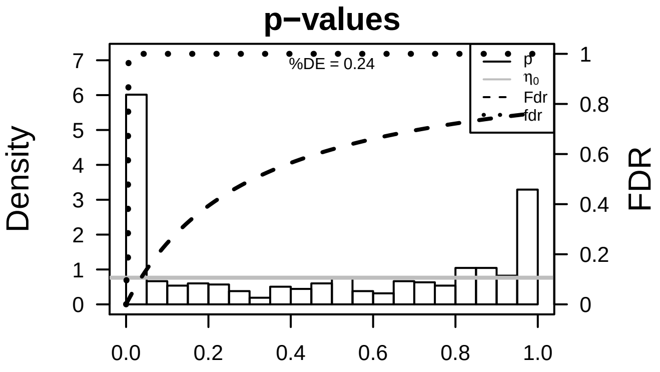

Supplement: Supplementary file 1 [file cells-09-00779-s001.zip › Supplementary materials/FileS3.zip!/FileS2/Hormones - Results/Summary Sheets - Samples/Zhewan.1.25.2..pdf]
